# Advanced Assistive Maintenance Based on Augmented Reality and 5G Networking

**DOI:** 10.3390/s20247157

**Published:** 2020-12-14

**Authors:** Sebastiano Verde, Marco Marcon, Simone Milani, Stefano Tubaro

**Affiliations:** 1 Department of Information Engineering, University of Padova, 35131 Padua, Italy; sebastiano.verde@dei.unipd.it (S.V.); simone.milani@dei.unipd.it (S.M.); 2 Dipartimento di Elettronica, Informazione e Bioingegneria, Politecnico di Milano, 20133 Milan, Italy; stefano.tubaro@polimi.it

**Keywords:** augmented reality, 5G real-time networking, assistive maintenance, edge computer processing

## Abstract

Internet of Things (IoT) applications play a relevant role in today’s industry in sharing diagnostic data with off-site service teams, as well as in enabling reliable predictive maintenance systems. Several interventions scenarios, however, require the physical presence of a human operator: Augmented Reality (AR), together with a broad-band connection, represents a major opportunity to integrate diagnostic data with real-time in-situ acquisitions. Diagnostic information can be shared with remote specialists that are able to monitor and guide maintenance operations from a control room as if they were in place. Furthermore, integrating heterogeneous sensors with AR visualization displays could largely improve operators’ safety in complex and dangerous industrial plants. In this paper, we present a complete setup for a remote assistive maintenance intervention based on 5G networking and tested at a Vodafone Base Transceiver Station (BTS) within the Vodafone 5G Program. Technicians’ safety was improved by means of a lightweight AR Head-Mounted Display (HDM) equipped with a thermal camera and a depth sensor to foresee possible collisions with hot surfaces and dangerous objects, by leveraging the processing power of remote computing paired with the low latency of 5G connection. Field testing confirmed that the proposed approach can be a viable solution for egocentric environment understanding and enables an immersive integration of the obtained augmented data within the real scene.

## 1. Introduction

Due to the growing cost of specialized technicians, the increasing complexity of maintenance work and the rising criticality of long machine downtime, Augmented Reality (AR) is playing an ever-growing role in advanced and remotely assisted maintenance [1,2]. In many cases, modern industrial service applications require specialized interventions, and apprenticeship programs are expensive and time-consuming. New employees need on-site oversight from more experienced technicians, which implies increasing costs and complicating the labor model, compromising service efficiency and effectiveness. Traditional actions taken by different societies are the following:

- dedicated systems for service technicians to communicate with one another and the back-office;
- access to data dashboards and detailed reports;
- employment of journeymen for on-boarding of new technicians.

Nowadays, however, these solutions alone are not enough: the whole system can be made more efficient by adopting newer models that enable the assistive guidance of experienced workers without being physically on-site, and deliver guidance to more apprentices at the same time. An effective back-office interaction with field employees is thus becoming a crucial point for an efficient maintenance policy. Moreover, thanks to emerging technologies such as AR helmets and 5G networking, traditional interactions by means of cellphones or work-specific messenger applications are overtaken by more immersive and interactive approaches.

**Figure 1:**
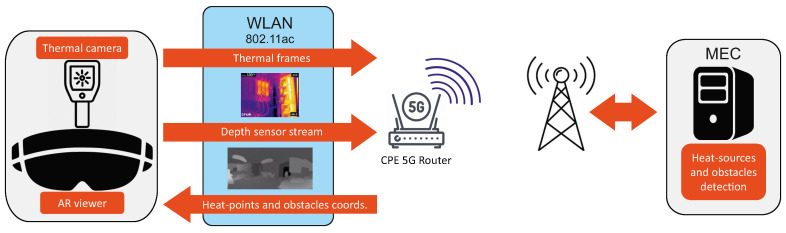
The proposed Advanced Assistive Maintenance framework. An AR viewer, equipped with a depth sensor and a thermal camera, sends multi-sensor information over an IEEE 802.11ac WLAN to a customer-premises equipment (CPE). The CPE is then connected through a 5G link to a Multi-access Edge Computer (MEC) that processes the received data to detect heat-sources and obstacles. Coordinates of detected points are sent back to the helmet and visualized in real-time. The same connection is also used for an interactive video call.

In this paper, we present a complete advanced assistive maintenance framework (Figure 1), deployed and tested in a real-world scenario. The on-site operator is provided with an AR helmet equipped with a thermal camera and a depth sensor. All these devices are connected in a WLAN based on the IEEE 802.11ac protocol and send data to a customer-premises equipment (CPE). The CPE provides a 5G connection to a remote control room, which hosts a Multi-access Edge Computer (MEC) that enables performing complex processing tasks for the egocentric vision system in real-time. A further remote call with a fully shared augmented reality environment was also implemented to allow an advanced support by an expert from a control room. The MEC approach represents in fact an affordable compromise to bring applications from centralized data centers in the cloud to the network edge, closer to the consumers and the data acquired by multiple sensors, and is acknowledged as the main brick to accomplish 5G 3GPP specifications [3].

The rest of the paper is organized as follows. Section 2 overviews the field of AR for egocentric vision systems. Section 3 frames the proposed system within the 5G panorama. Section 4 and Section 5 describe the algorithms developed to process the acquired 3D scene and integrate the information from heterogeneous sensors (depth and thermal cameras), respectively. Section 6 illustrates the prototyping and testing phase, along with a discussion of the obtained results. Finally, Section 7 concludes the paper and draws the overall conclusions.

## 2. AR for Egocentric Vision

The first Head-Mounted Displays (HMDs) date back to the 1960s, when the head tracking capability was first introduced by Sutherland [4]; then their use grew continuously, first in the Virtual Reality (VR) world for networked applications such as DIVE [5] or MASSIVE [6] and then in the 1990s, for the networked AR systems such as Studierstube [7] or Shared Space [8] (further historical details can be found in [9]).

Nowadays, we dispose of a wide variety of VR and AR devices, from cheap, smartphone-based ones, such as the papercraft FOV2GO from the University of Southern California [10], to more advanced and professional devices [11] such as Microsoft HoloLens, Magic Leap One and Google Glass. In addition to assistive maintenance [1,2], AR has proven its effectiveness in a wide range of applications, such as training for high-precision tasks [12], indoor navigation in large buildings (such as hospitals, airports and train stations) [13], education [14], image-guided surgery [15] and telemedicine [16].

Within the different features of AR for assistive maintenance, *telepresence*, i.e., the possibility to interact with a remote specialist along with real and virtual objects, is among the most exciting and requested capabilities. Telepresence can be carried out in different forms, from the old and simplest voice call to the most advanced *telexistence*, where a full holographic representation of the remote assistant is able to interact in real-time with virtual objects in the real scene. Of course, the higher the interaction complexity, the larger the required amount of exchanged data. Furthermore, the transmission latency must be as low as possible to ensure an immersive and realistic interaction.

The reasons for an unnatural and, in some cases, disturbing fusion of real and virtual elements in HMDs are to be found in the following: delays between the helmet’s movement and the refresh of the virtual scene; oversimplified 3D mesh surfaces; wrong ray-traced illumination of virtual objects. Recent smartglasses, such as Microsoft HoloLens, cope well with these aspects thanks to the integration of the internal Inertial Measurement Unit (IMU) with the on-board processing of multiple cameras and depth-cameras in a Simultaneous Localization and Mapping (SLAM) approach [17]. The user’s head position is continuously tracked with high accuracy so that no delay or misplacement in the rendering of virtual objects is noticeable. However, an additional physical cause of disturbing effects is related to the imperfect eyes trick induced by images projected on the holographic lenses. The human visual system allows for the perception of a three-dimensional world from plain and ambiguous stereo images obtained from the eyes. There are different theories on how this is accomplished; one of the most credited is based on the *Cue Theory* [18], where 10 depth cues interact with lighting and scene structure in order to provide a complete three-dimensional understanding [19]:

- binocular disparity;
- binocular convergence;
- accommodative focus;
- atmospheric haze;
- motion parallax;
- linear perspective and foreshortening;
- occlusion;
- height in the visual field;
- shading;
- texture gradient.

AR allows to trick our visual system into merging virtual objects seamlessly with the real environment by acting on some of the aforementioned cues. Still, HMDs suffer from a series of drawbacks due to forcing the user to a fixed accommodative length of the crystalline lens and to the limited field of view, causing AR to feel unnatural. Furthermore, according to [20,21,22], the aforementioned problems cause an underestimation of the egocentric depth, at least in the user’s neighborhood, with a mean error ranging from −0.4 to −0.8 meters for distances between 3 and 7 m [20]. This last aspect could represent a serious hazard in industrial environments.

The proposed system deals with some of the most common purposes of AR assistive maintenance:

- indoor navigation;
- remote interaction with a remote specialist;
- contact with hot surfaces avoidance;
- obstacle avoidance.

For all of them, a wrong perception of the distances of virtual objects in the HMD could result in a faulty service and introduce further risks to the operator’s safety. According to [23,24], it is possible to limit the erroneous distances perception by applying a linear rescaling of depth maps acquired by the depth camera, just for the egocentric AR visualization system. Virtual objects placed in this stretched frame of reference are then perceived as properly overlapped to their real counterpart. The transformation links the new depth $d^{\prime }$ to the original one *d* according to (1):

(1) $$d^{\prime }=\frac{d+0.1}{0.9}$$

The description of the indoor navigation task is kept out of this paper, since it is based on the approach provided in [25]: the 3D point clouds and surfaces acquired by HoloLens provide an accurate indoor localization, from which navigation is obtained by visualizing holographic AR signage on the HMD. No audio indications were considered, due to the possible occurrence of noisy environments in industrial plants.

## 3. 5G Networking

The fifth generation of mobile communication networks (5G) sets some stringent requirements related to ultra-low-latency, ultra-responsive and ultra-reliable services. The only way of accomplishing these requests is to distribute the computational power from the central cloud to a Multi-access Edge Computing (MEC). The MEC approach allows moving storage and relevant computation tasks closer to the access network, reducing the traffic volume and achieving real-time performance for AR applications, together with context-awareness and enhanced privacy protection [3,26].

AR and VR technologies are expected to be among the most relevant applications of the 5G/MEC paradigm, with a predicted market of $179 billion by 2021 [27]. On the other hand, this implies a large growth in network traffic. One of the most widely proposed countermeasures consists in local caching at the Edge [28], which comes particularly handy in an assistive maintenance scenario as it allows to keep frequently accessed documents, 3D models and holographic videos directly on the MEC, reducing the load in backhaul links and cloud servers.

Caching also reduces the end-to-end latency, allowing in our case to upload 3D mesh surfaces at an average speed of 156 MB/s, equivalent to approximately 1.5 M triangles/s. Assuming an average environment mapping composed of 20 K triangles (empirical measurements are reported in Table 1), an entire new map can be uploaded (e.g., while the operator enters a new room) in less than 20 ms. Entering different rooms, along with the associated holographic augmented elements, is perceived seamlessly by the user as the whole procedure is carried out within the HoloLens’ framerate. It is worth mentioning that the WiFi transmission between the 5G modem/router and HoloLens is performed with a 802.11ac connection in the 5 GHz band [29], whose bandwidth is large enough to not introduce any significant delay.

Once the HMD enters a new room, it starts exploring changes with respect to the previously stored map and periodically transmits the new 3D mesh back to the MEC, where the Unity development platform [30] runs the analyses described in Section 4. Together with HoloLens’ data, RGB and thermal images (Section 5) are transmitted to the MEC as well, but their incidence is negligible (around 6.5 MB/s) with respect to the overall data stream.

## 4. Scene Processing

The core feature of our assistive maintenance framework consists of a scene understanding software that: (i) makes use of depth sensors to acquire 3D data from the environment; (ii) processes such data on an edge computing platform to detect and localize potentially dangerous objects; (iii) highlights these objects in real-time to the operator by projecting 3D holograms onto the HMD.

The HMD adopted for deploying the system is Microsoft HoloLens. These AR glasses are equipped with a Time-of-Flight (ToF) sensor that constantly acquires depth information from its surroundings and progressively builds and updates a 3D representation of the environment, in the form of a triangular mesh. The on-board computational power is, however, not sufficient to perform real-time analysis on the recorded 3D data; therefore, the environmental mesh is transmitted over a 5G network to an MEC, where the whole processing task is carried out by a set of specifically designed algorithms (implemented in Unity). The output consists of a set of obstacle coordinates, which are sent back to the AR glasses and visualized on the integrated holographic display.

Providing a formal definition for obstacles is no trivial task. Generally speaking, we may associate the concept of an obstacle to an object impeding or limiting the execution of a given action. In the specific case of on-field assisted maintenance, we chose to narrow our focus to isolated objects on the ground (or hanging from the ceiling) that stand in the operator’s way and, if unnoticed, may hamper its movements or result in potential security threats. Given a 3D mesh of the environment, we call *obstacle* any part of the mesh within a predefined size range that results disconnected from the scene once removed the floor (or ceiling) plane. The following paragraphs provide a detailed description of the algorithms developed to preprocess the environmental mesh, extract the ground-plane and localize the isolated components.

### 4.1. Mesh Simplification

AR devices typically acquire significant amounts of 3D data, with environmental meshes easily counting tens (or even hundreds) of thousands of vertices. While the actual number of bits remains limited to a few megabytes and thus does not impair the required low latency, the computational complexity on the MEC side may increase rapidly and non-linearly with the number of 3D points. To prevent the formation of a bottleneck at the edge computing side, we added a preprocessing step that simplifies the received mesh before feeding it to the obstacle detection module.

Mesh simplification consists of a class of algorithms that transform a given mesh into an approximated version with fewer vertices and faces. A variety of approaches have been proposed by the scientific community, addressing different application-specific quality criteria [31]. Our implementation falls within the *vertex clustering* subclass [32], which is considered to be among the most efficient, once paying the price of removing small-shape details. While the spatial resolution of the original mesh is fundamental to the HMD for an accurate positioning of holograms in the real world, a rougher representation of the environment is more than enough for the task of detecting macroscopic obstacles.

In Unity notation, a triangular mesh is an object M=(V,T), where *V* is an array of 3D vertices and *T* is a concatenation of triplets of vertex indices, denoting triangular faces. The proposed algorithm receives a triangular mesh, *M*, and outputs a simplified version of the same, herein referred to as $M^{\prime }=\left(V^{\prime },T^{\prime }\right)$, with respect to a given quantization parameter, δ.

The simplification procedure starts with quantizing the three-dimensional space into a lattice of cubes with δ-sized edges. The algorithm then scans through the vertices in *V*, retaining only one point per cube and keeping track of the discarded ones. Finally, new triangles are built by looping over *T* and remapping the indices of the removed vertices into those of the retained ones. A pseudocode representation is provided in Algorithm 1. Note that this implementation produces redundant copies of some triangular faces, which are deliberately *not* removed from $T^{\prime }$, since they do not impair Unity’s performance and, therefore, would result in a waste of computational time.

The simplified mesh $M^{\prime }$ is now passed to the ground-plane extraction step.
**Algorithm 1** Mesh simplification algorithm. Requires: *V*, array of 3-D vertices; *T*, concatenation of triplets of vertex indices, denoting triangles; δ, spatial quantization step. 1: **procedure**
Mesh Simplify (M=(V,T),δ) 2:   n←0▹ New number of vertices 3:   **for**
*i* in range of |V|
**do** 4:     $x,y,z\leftarrow \lfloor V\left[i,:\right]\cdot \delta^{-1}\rfloor$▹ Quantized vertex coordinates 5:     **if** no vertex quantized to (x,y,z) yet **then** 6:      $V^{\prime }\left[n\right]\leftarrow V\left[n\right]$ 7:      previous[x,y,z]←n 8:      old2new[i]←n 9:      n←n+1 10:    **else** 11:     old2new[i]←previous[x,y,z] 12:     **end if** 13:   **end for** 14:   **for**
*j* in range of |T|**do**▹ Make new triangles 15:     $T^{\prime }\left[j\right]\leftarrow old2new\left[T\left[j\right]\right]$ 16:   **end for** 17:   **return**
$M^{\prime }=\left(V^{\prime },T^{\prime }\right)$▹ Simplified mesh 18: **end procedure**

### 4.2. Ground-Plane Extraction

Estimating the coordinates of the ground-plane represents a critical step for obstacle detection, as it defines the elevation level from which to start detecting isolated mesh components. The algorithm for this task was designed around the specific features of the employed HMD.

HoloLens’ world coordinates are initialized to (x,y,z)=0 at the point in space where its operating system is booted. Also, the embedded IMU allows the device to orientate its *y*-axis along the direction of the gravity vector. Therefore, the ground-plane is always perpendicular to the *y*-axis (under the assumption of a non-skewed floor) and fully described by the one-parameter equation $y=y_{G}$.

**Figure 2:**
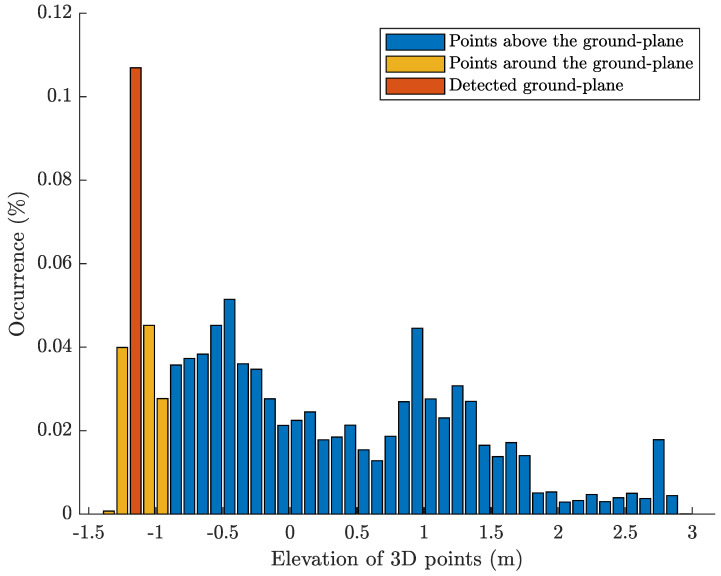
Histogram of vertex elevations in the acquired environmental mesh. The ground–plane is estimated as the middle–point of the most numerous bin (10 cm wide) among the first 10% of the mesh’s elevation range.

Once the MEC receives the first (simplified) mesh from the AR device, $y_{G}$ is estimated in real-time by analyzing the histogram of vertices’ *y*-coordinates (Figure 2). Given that the depth sensor cannot detect any point below the ground-plane (except for overestimation errors), and with the floor being the largest flat surface in the bottom part of the scene, we expect to observe a considerably higher bin among those related to the lowest elevations.

The number of histogram bins is set to

$$k=\left\lceil \frac{y_{\max}-y_{\min}}{\delta }\right\rceil ,$$

where $y_{\max}$ and $y_{\min}$ are the *y*-coordinates of the extreme vertices of the mesh along the vertical dimension.

Let $m_{y}$ be the count of the histogram bin associated to the elevation interval

$$\left[y-\frac{\delta }{2},\;y+\frac{\delta }{2}\right]$$

and let

$$Y_{L}=\left[y_{\min},\;y_{\min}+p\left(y_{\max}-y_{\min}\right)\right]$$

be a predefined range of elevations, from the lowest vertex $y_{\min}$ up to a given percentage *p* of the full span. The ground-plane parameter $y_{G}$ is estimated as

(2) $$y_{G}=\underset{y\in Y_{L}}{\arg\max}\;m_{y}$$

Once the elevation of the ground-plane is calculated, the algorithm removes any vertex *v* from the environmental mesh such that $y_{v}\leq y_{G}+\delta$. Every object lying on the floor, therefore, becomes an independent submesh.

### 4.3. Objects Detection

The problem of detecting isolated submeshes within the global mesh may be reinterpreted as a graph theory problem, leveraging the concept of connected components.

Let M=(V,T) be a mesh object as defined in Section 4.1. One can build an undirected graph G=(V,E) where every vertex in *G* corresponds to a vertex in *M*; an edge e∈E exists between two vertices if the same are connected by a triangle side in *M*. By doing this, isolated submeshes in *M*’s domain translate into connected components in *G*’s one.

**Definition** **1.** *A connected component (or simply component) of an undirected graph is a subgraph in which any two vertices are connected to each other by a path of edges, and which is connected to no additional vertices in the supergraph.*

Computing all the components of an undirected graph is achievable in linear time, O(|V|+|E|), using either breadth-first or deep-first search [33]. While looping through all vertices in *V*, a new search is started whenever the loop encounters a vertex *v* that has not yet been included into a previously found component. The search will then find the entire component containing *v* before returning.

Our implementation of the algorithm (using deep-first search) is provided in Algorithm 2. It is straightforward to see how this approach returns in fact *every* connected component of the mesh-graph: along with actual objects and obstacles, walls themselves, for instance, constitute a component. We observed, however, that uninteresting outcomes can be easily filtered out by an adjustable threshold on components’ dimensions (see Section 6.2).

The set of identified obstacles is now sent back over the 5G link to the AR device, in the form of a list of 3D coordinates, allowing for such points to be highlighted to the operator by positioning holograms corresponding to their spatial locations.
**Algorithm 2** Connected components retrieval algorithm for undirected graphs. Requires: G=(V,E), undirected graph. Ensures: *S*, set of *G*’s connected components. 1: **procedure**
Components(G=(V,E)) 2:   S←∅ 3:   **for**
v∈V
**do** 4:       **if**
*v* not visited **then** 5:        C←∅▹ New component 6:        RecursDFS(v,C) 7:       **end if** 8:   **end for** 9:   **return**
*S* 10: **end procedure**  11: **function**
RecursDFS(v,C)▹ Deep-First Search 12:  mark *v* as visited 13:  C←C∪{v} 14:  **for**
*x* adjacent to *v*
**do** 15:   **if**
*x* not visited **then** 16:    RecursDFS(x,C) 17:   **end if** 18:  **end for** 19: **end function**

## 5. Thermal Sensing Integration

The additional challenge of our framework consisted in the integration of an external thermal sensor cooperating with the scene understanding software in order to detect and signal the presence of harmful heat sources. To do so, we installed a commercial thermal camera on the helmet and calibrated it with the AR device’s embedded depth sensor.

We approached the problem of cross-calibrating the two-sensor system as a stereo calibration [34]. Since ToF sensors allow the acquisition of reflectivity information alongside with depth (Section 6) and the adopted thermal camera also embeds a standard RGB one, by treating reflectivity maps as gray-scale images, we can straightforwardly calibrate the system using a checkerboard pattern [35].

The calibration parameters we are interested in are as follows:

- $K_{D}$, $K_{T}$, the *intrinsic* parameters of the two cameras;
- R, t, the *extrinsics*, i.e., the rototranslation of one camera with respect to the other;
- F, the *fundamental matrix* of the two-view system.

From these, we can write down the projective models for the two cameras as

(3) $$x_{D}=P_{D}X=K_{D}\left[I\;|\;0\right]\;X,$$

(4) $$x_{T}=P_{T}X=K_{T}\left[R\;|\;t\right]\;X,$$

where $X={\left(X,Y,Z,1\right)}^{⊤}$ is a real-world 3D point in homogeneous coordinates; x=(x,y,1)⊤ is the projected point on the image plane; $P_{D}$, $P_{T}$ are the respective *camera matrices*. Note, that we are adopting the *center of projection* of the depth camera as the origin of our world coordinates.

**Figure 3:**
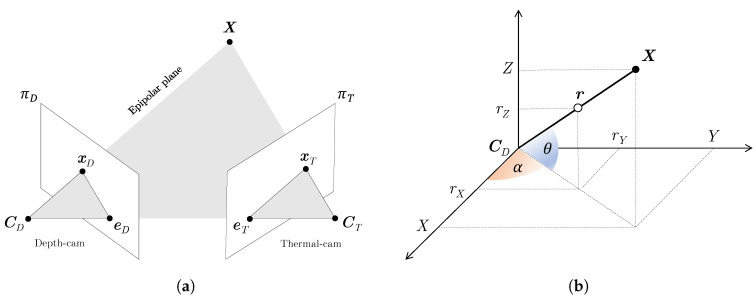
Epipolar geometry relations between the calibrated depth and thermal cameras (**a**) and 3D coordinates reconstruction of a given point X with known depth value (distance from the center of projection of the depth camera, $C_{D}$) and optical ray r (**b**).

The two-sensor system is now described by the *epipolar geometry* [36], which models the relations between the observed 3D points in the scene and their projections onto the two camera planes, namely $\pi_{D}$ and $\pi_{T}$ (Figure 3a). In particular, given a generic world-point X, the plane identified by X and the two centers of projection, $C_{D}$, $C_{T}$, is called the *epipolar plane*. The projections of X onto $\pi_{D}$ and $\pi_{T}$, namely $x_{D}$ and $x_{T}$, lie themselves on the epipolar plane.

These geometric relations are utilized in the following paragraph to describe the algorithm designed to calculate the 3D coordinates of a heat-point given its 2D projection on the thermal camera plane. We leave the criterion by which to select such heat-points out of the discussion, as it is usually application-dependent, and we focus instead on the procedure to be applied for a generic given pixel on the thermal image.

### 5.1. Heat-Point Triangulation

Let ${\hat{x}}_{T}$ be a point on $\pi_{T}$. Our aim is to calculate the 3D point $\hat{X}$ that gets projected into ${\hat{x}}_{T}$. To reconstruct the 3D position of a point in $\pi_{T}$, we first need to find its corresponding point ${\hat{x}}_{D}$ on $\pi_{D}$. From epipolar geometry, we know that such a point lies on the *epipolar line*, i.e., the projection on $\pi_{D}$ of the optical ray through $C_{T}$ and $\hat{X}$. Given the fundamental matrix, F, obtained by calibration, the parametric representation of the epipolar line in homogeneous coordinates is

(5) $$l_{D}=F^{⊤}{\hat{x}}_{T}$$

For each point $x_{D}=\left(x_{D},y_{D},1\right)$ on line $l_{D}$, we can now invert the relation in (3),

(6) $$r=K_{D}^{-1}x_{D}$$

obtaining the directing vector of the optical ray $r={\left(r_{X},r_{Y},r_{Z}\right)}^{⊤}$.

Since each point in $\pi_{D}$ is associated to a depth value *d*—equal to the distance of the 3D point from the center of projection—we are able to unequivocally reconstruct X’s coordinates through trigonometric relations. From Figure 3b, one can derive the following identities:

(7) $$\tan\theta =\frac{r_{Z}}{\sqrt{r_{X}^{2}+r_{Y}^{2}}}\;,\;\tan\alpha =\frac{r_{Y}}{r_{X}}$$

and hence the 3D coordinates of X are given by

(8) $$\tilde{X}=\left[\begin{array}{c} d\cos\theta \cos\alpha \\ d\cos\theta \sin\alpha \\ d\sin\theta \end{array}\right]\;,\;X=\left[\begin{array}{c} \tilde{X}\\ 1 \end{array}\right]$$

where $\tilde{X}$ denotes X in Cartesian coordinates.

Given the set of reconstructed X points, it is possible to reproject them on $\pi_{T}$ using (4), obtaining a set of $x_{T}$ points lying on the epipolar line $l_{T}$. By taking account for reprojection errors, we can finally select $\hat{X}$ among the reconstructed points as the one whose projection $x_{T}=P_{T}X$ minimizes the distance from the original point ${\hat{x}}_{T}$ on the thermal camera plane,

(9) $$\hat{X}=\underset{X}{\arg\min}\;∥P_{T}X-{\hat{x}}_{T}∥$$

Note that the total number of operations required by the algorithm is proportional to the number of points on the epipolar line, which is quadratically less complex than searching through the whole image. This feature helps speed up the triangulation of a 3D point dramatically, allowing a real-time response from the detection of a heat-point on the thermal plane to the reprojection and signaling of the same on the AR viewer.

## 6. Prototyping and Testing

**Figure 4:**
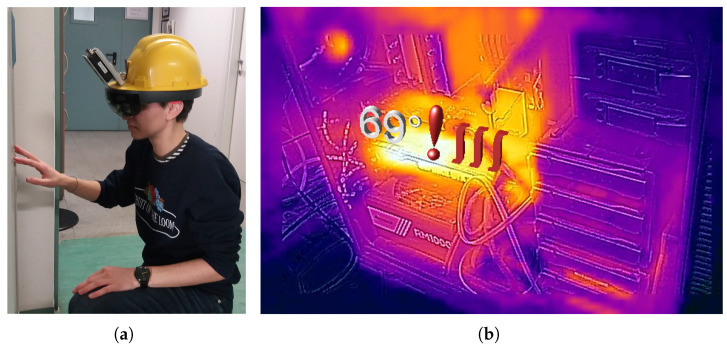
Prototype helmet equipped with AR smartglasses and thermal camera (**a**); thermal image combined with the warning hologram reprojected in the HMD display (**b**).

Assessing the performance of the proposed assistive maintenance framework first required the realization of a prototype. The employed AR device (Microsoft HoloLens) was installed on the bottom end of a safety helmet (see Figure 4a) of the kind that is usually adopted for on-field maintenance operations. The helmet was also equipped with a FLIR AX8 thermal camera, calibrated along with HoloLens’ depth sensor as a two-view stereo system (see Section 5.1).

**Figure 5:**
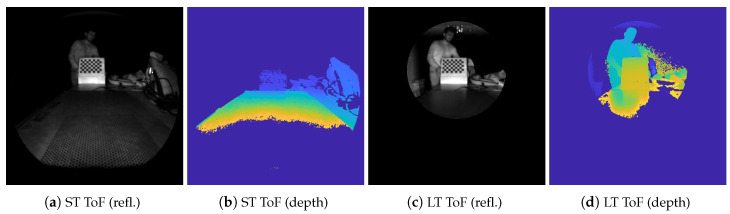
Example frames from HoloLens’ ToF depth camera. The sensor acquires 450 × 448 depth and reflectivity maps, in short-throw (ST), i.e., up to ∼1 m, and long-throw (LT), i.e., up to ∼3 m.

Microsoft HoloLens embeds a ToF depth sensor with two infrared illuminators, namely *short-throw* (up to ∼1 m) and *long-throw* (up to ∼3 m), operating on different fields of view and acquiring depth and reflectivity information simultaneously with a resolution of 450 × 448 pixels (see Figure 5 for sample frames).

**Figure 6:**
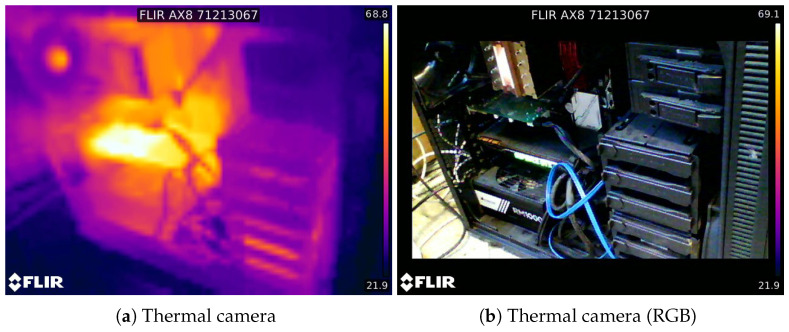
Example frames from the FLIR AX8 thermal camera. The sensor acquires 80 × 60 thermal maps and 640 × 480 RGB images.

FLIR AX8 thermal camera acquires 80 × 60 thermal images with a temperature range from −10 ${}^{\circ }$C to 150 ${}^{\circ }$C and a field of view (FOV) of 48${}^{\circ }$× 37${}^{\circ }$; it acquires standard RGB images as well with the same FOV and a resolution of 640 × 480 pixels (see Figure 6 for sample frames).

### 6.1. Testing Environment

Obstacle detection tests were conducted with four human testers inside a Vodafone Base Transceiver Station (BST) to simulate real-case maintenance tasks.

Each tester was located inside the BTS wearing the prototype AR helmet. HoloLens was connected wirelessly to the CPE and two computers were connected to the same network: a terminal for the remote assistant in the control room; the MEC, hosting the developed algorithms for obstacle detection. Two applications were running on HoloLens: a video call app, allowing the communication between the on-field tester and the remote assistant; the obstacle visualization app, which receives 3D coordinates from the MEC and projects holograms onto the AR viewer in correspondence of the spatial locations of the detected objects. Both on-board and MEC applications were developed in Unity.

Thermal sensing integration tests were conducted during the *Milano Digital Week 2019*—held in March 2019, in Milan, Italy—on the occasion of the first presentation of the prototype. Tests were carried out by utilizing an incandescent lamp that was being detected by the thermal camera and whose coordinates were correctly estimated by the algorithm described in Section 5.1 and displayed by a 3D holographic marker on the AR viewer. The choice of which points to reproject from the thermal camera plane for 3D triangulation was done according to a temperature threshold of 60 ${}^{\circ }$C.

### 6.2. Obstacle Detection Results

For the purpose of implementing a reliable framework for assistive maintenance, we addressed a particular focus to the real-time response of the obstacle detection system. In order to evaluate it, we used as Key Performance Indicator (KPI) the mesh processing time in relation to the number of points and triangles.

We set the quantization parameter for mesh simplification (also used as the histogram bin size for ground-plane extraction) to δ=10 cm (see Section 4.1). The percentage of elevations used to detect the histogram peak associated to the ground-plane was set to p=10%. Among the retrieved mesh-graph components, we considered obstacles to be those lying within 1 m from the floor-plane.

Table 1 reports some results on the measured computation times for the tasks of mesh simplification, ground-plane extraction and object detection, in relation to the number of vertices (before and after reduction), triangles and detected obstacles. Measurements were collected sequentially, as the mesh acquired by the AR device grows while the environment is being explored. From these results, it is evident how mesh simplification helps to stabilize the total computation time: while the number of vertices in the non-processed mesh grows from thousands to tens of thousands, the overall time required for processing the scene remains below 100 ms, on average. Additionally, the fraction of computation time needed for detecting the mesh-graph components is most of the time negligible. It may surprise to observe that the number of detected obstacles do not follow a strictly non-decreasing trend, but this is in fact perfectly in line with the way HoloLens creates and refines environmental meshes: since new acquisitions are constantly being incorporated into the current model, it happens quite often to see an object being first acquired as multiple disconnected components and then being completed within the next few frames. The bandwidth and the latency of the 5G network, together with their counterpart in the 802.11ac WLAN, did not introduce any delay both in the real-time bidirectional data streaming to and from the HoloLens. An exhaustive 5G transmission speed and latency tests were not performed since the whole use-case was tested in an experimental 5G setup with limited performances with respect to the final operative deployment; however, the scene processing together with the remote call interaction and the proper hologram placement was always seamless and no disruptive delays occurred.

### 6.3. Cross-Sensor Calibration

Cross-calibration of depth and thermal cameras was carried out with a standard 7×9 checkerboard pattern, 26.5 mm per square, moved at a distance of about 1 m from the prototype helmet. Pictures of the pattern were taken using the depth sensor’s reflectivity camera and the thermal sensor’s RGB camera. Calibration was performed in accordance with the pipeline established by [35]: (i) estimate the intrinsic and extrinsic parameters in closed-form solution; (ii) refine all parameters, including lens distortion, with a non-linear minimization. We used the implementation available in Matlab R2019a.

**Figure 7:**
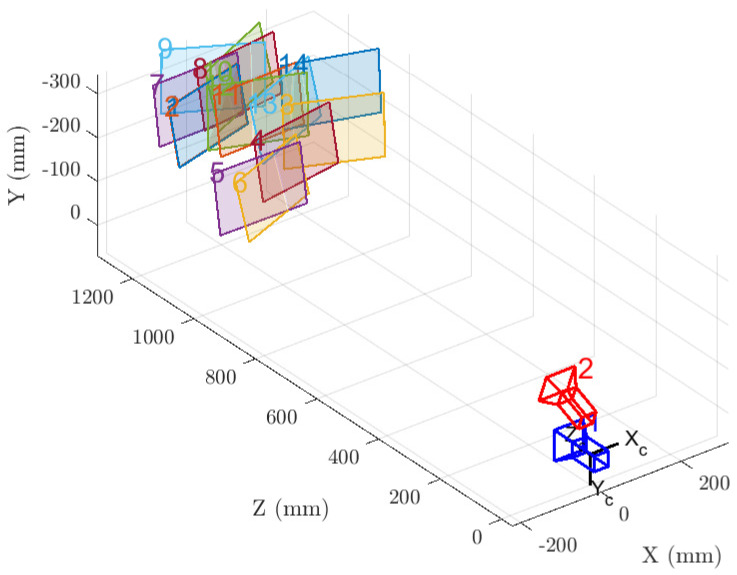
Extrinsic parameters visualization for the cross–calibration of ToF–sensor (blue) and thermal camera (red) using a moving checkerboard pattern, combining ToF reflectivity with thermal RGB information.

The procedure returned the following reprojection errors, in unit of pixels:

$$\begin{array}{ccc} \epsilon_{D} & = & 0.1571,\\ \epsilon_{T} & = & 0.2542, \end{array}$$

with an average error $\bar{\epsilon }=0.2056$. A visualization of the estimated extrinsic parameters is provided in Figure 7, where one can verify how the thermal camera (in red) correctly resembles its collocation on top of the helmet, above the AR viewer and the associated depth camera (in blue).

Given the obtained sub-pixel reprojection error, we can conclude that a standard stereo calibration procedure is suitable as well for cross-calibrating heterogeneous sensors, combing visible and reflectivity information.

### 6.4. Remote Assistance

**Figure 8:**
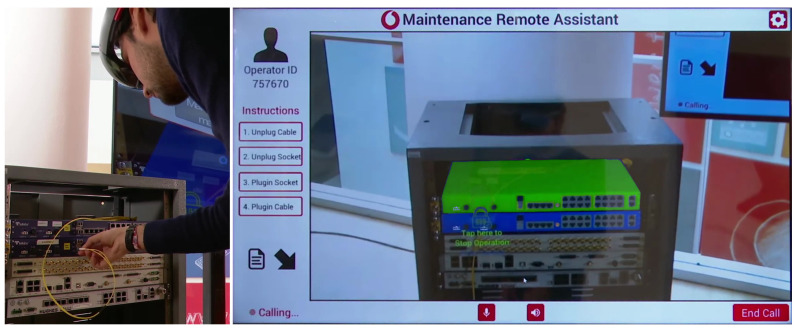
A snapshot of a remote call with real-time augmented reality interaction: (**left**) the on-field operator follows the instructions provided by the remote assistant; (**right**) the monitor of the remote assistant displays a replica of the AR scene as seen by the operator; the devices involved in the maintenance intervention are highlighted by superimposing and visualizing the respective holograms on the HMD.

In view of a fully-capable on-field adoption, the system was also equipped with an augmented reality remote assistance service that allows the on-field operator to interact with the remote expert. The two share a real-time audio and video streaming based on *Skype for HoloLens*^®^ with virtual objects and 3D markers in overlay, as depicted in Figure 8.

## 7. Conclusions

Augmented reality and 5G networking are paving the way to a whole new set of possibilities in the progressive digitization of modern industries. Remote assistive maintenance based on egocentric environment-aware devices represents a viable solution to improve workers’ safety in dangerous locations and the communication efficiency between on-site operators and remote assistants.

In this paper, we presented a fully-developed assistive maintenance system based on a wearable AR device equipped with environment-understanding and thermal sensors. A set of algorithms was specifically designed to detect potentially dangerous objects and heat-sources, while the holographic viewer enables the visualization of such information directly in the operator’s field of view. Finally, deploying the analysis software on an edge computing platform connected to the device via a 5G link, allows for all the required processing to be performed in real-time.

## Figures and Tables

**Table 1:** Measured computation times for scene-processing tasks, namely: mesh simplification, ground-plane extraction and objects detection (via connected components retrieval). The acquisition is sequential: the number of mesh vertices (first column) grows as the environment is being explored by the Head-Mounted Display (HDM). Thanks to mesh simplification, the overall computation time remains stable on average as the mesh size increases.

Mesh Parameters				Computation Time			
No. Vertices	No. Triangles	No. Reduced Vertices	No. Detected Obstacles	Mesh Simplif. (ms)	Ground-Plane (ms)	Components (ms)	Total (ms)
1475	2414	518	1	25	8	6	39
4855	7656	1419	7	52	20	10	82
7928	12,764	2375	10	42	29	<1	81
9344	15,109	2914	10	33	32	<1	75
10,014	16,444	3054	14	39	35	<1	88
19,949	33,869	6164	9	64	61	1	125
20,125	20,125	1828	6	38	60	<1	98
